# Etiological profile of male urethritis and genital ulcer disease and antimicrobial susceptibility of *Neisseria gonorrhoeae* in Southeastern Brazil

**DOI:** 10.1016/j.bjid.2026.105823

**Published:** 2026-05-11

**Authors:** Felipe Barufaldi, Maria Luiza Bazo, Jessica Motta Martins, Lis Aparecida de Souza Neves, Henrique Ciabotti Elias, Renata Karina Reis, Marcela Antonini, Rodrigo de Carvalho Santana

**Affiliations:** a Universidade de São Paulo, Faculdade de Medicina de Ribeirão Preto, Ribeirão Preto, SP, Brazil; b Universidade Federal de Santa Catarina, Florianópolis, SC, Brazil; c Secretaria Municipal de Saúde de Ribeirão Preto, Ribeirão Preto, SP, Brazil; d Universidade de São Paulo, Escola de Enfermagem de Ribeirão Preto, Ribeirão Preto, SP, Brazil

**Keywords:** Urethritis, Sexually transmitted infections, *Neisseria gonorrhoeae*, Antimicrobial resistance

## Abstract

**Background:**

Sexually Transmitted Infections (STIs) are major causes of male urethritis and genital ulcer disease, with increasing concern about antimicrobial resistance, particularly in *Neisseria gonorrhoeae*. This study aimed to describe the etiological profile of male urethritis and genital ulcer disease and to evaluate the antimicrobial resistance profile of *Neisseria gonorrhoeae* isolates in a medium-sized Brazilian city.

**Methods:**

To address the study objectives, a descriptive study was conducted among men aged 18-years or older presenting with urethral discharge and/or genital ulcers at STI reference services in Ribeirão Preto, São Paulo State, Brazil. Urethral and ulcer swabs were analyzed using molecular assays to identify etiological agents. Antimicrobial susceptibility testing of *N. gonorrhoeae* isolates was performed by determining minimum inhibitory concentrations according to international standards.

**Results:**

A total of 144 samples were obtained from 134 men (mean age: 29-years), including 126 urethral samples and 18 genital ulcer disease samples. Neisseria *gonorrhoeae* was detected in 108 urethral samples (85.7 %), while *Chlamydia trachomatis* was identified in 45 (35.7 %), frequently in coinfections. Among genital ulcer disease cases, herpes simplex virus type 2 was detected in 7 cases (38.9 %), *Treponema pallidum* in 6 (33.3 %), and no pathogen was identified in 4 cases (22.2 %). Antimicrobial susceptibility testing was successfully performed in 95 Neisseria *gonorrhoeae* isolates, showing resistance rates of 71.7 % to ciprofloxacin, 43.5 % to tetracycline, and 26.1 % to penicillin, with complete susceptibility to ceftriaxone and cefixime. Coinfection with *Chlamydia trachomatis* and *Neisseria gonorrhoeae* occurred in 35 samples (28 %).

**Conclusions:**

Neisseria *gonorrhoeae* and *Chlamydia trachomatis* are the leading causes of male urethritis, whereas herpes simplex virus type 2 and *Treponema pallidum* predominate among cases of genital ulcer disease. The high prevalence of ciprofloxacin resistance reinforces the need for continuous antimicrobial resistance surveillance and supports current guidelines recommending ceftriaxone as first-line therapy.

## Introduction

Sexually Transmitted Infections (STIs) are a significant global public health concern. With over 720 million new STI cases and >1.6 million new HIV infections recorded in 2021, regions such as sub-Saharan Africa and Latin America are particularly affected. Despite minor fluctuations in non-HIV STI rates, overall case numbers are rising due to population growth and sustained transmission. Untreated STIs can result in infertility, adverse pregnancy outcomes, and a greater risk of HIV transmission.^1^ Untreated STIs can result in infertility, adverse pregnancy outcomes, and a greater risk of HIV transmission.^2^

Male urethritis and genital ulcer disease are consistent STI symptoms. Urethritis is mainly caused by *Neisseria Gonorrhoeae* (NG) or *Chlamydia Trachomatis* (CT), while genital ulcers primarily result from Herpes Simplex Virus (HSV-2) or *Treponema Pallidum* (TP). Accurate diagnosis is often hindered by limited laboratory resources.

In Brazil, although laboratory-based diagnostic methods, including molecular assays, have increasingly been incorporated into national STI management protocols, syndromic management remains the main approach in everyday clinical practice, and etiological testing is still underutilized in many healthcare settings. In this situation, monitoring antimicrobial resistance in NG is particularly important, considering the global increase in resistant strains.

This study aimed to clearly identify the causes of male urethritis and genital ulcer disease among patients at STI reference services in Ribeirão Preto, southeastern Brazil, and to detail the antimicrobial resistance patterns of NG isolates using laboratory-based surveillance methods.

## Methods

### Study design, participants, and recruitment

To achieve these aims, a descriptive study was conducted at three STI reference clinics in Ribeirão Preto, São Paulo, Brazil. Men aged 18-years or older who presented with urethral discharge and/or genital ulcers and sought care spontaneously at the participating clinics were invited to participate. Exclusion criteria included lack of prior sexual activity, suspected sexual abuse, complicated urogenital infections, and current use of systemic antimicrobial agents at the time of sample collection. Participants were recruited through convenience sampling between October 2018 and January 2021. Other variables such as sexual behavior, number of partners, condom use, and HIV status were not considered. This study was conducted as part of the Brazilian Sentinel Surveillance Program for Antimicrobial Resistance in Neisseria gonorrhoeae (SenGono Project).

### Sample collection and molecular testing

Urethral discharge and genital ulcer specimens were collected using sterile swabs following standardized procedures. Urethral samples were obtained with swabs placed in Amies transport medium with charcoal, and the samples were transported to the local supporting laboratory within 6-hours of collection. Samples intended for molecular analyses were stored at 2–8 °C at the collection sites and later transported to reference laboratories in accordance with established project protocols. Genital ulcer specimens were collected using the Universal Transport Medium RT system (UTM™–RT; Copan, USA). In patients with multiple ulcers showing different clinical features, separate swabs were taken, prioritizing the most prominent lesions. Molecular assays for etiological identification were performed at the Laboratory of Molecular Biology, Microbiology, and Serology (LBMMS/UFSC), using validated molecular methods as part of the diagnostic workflow.

After DNA extraction, the Allplex STI Essential Assay real-time multiplex PCR kit (Seegene, Seoul)14 was used to detect the following pathogens in urethral discharge samples: CT, MG, MH, NG, TV, UP, UU. In genital ulcer samples, the Allplex Genital Ulcer Assay kit (Seegene, Seoul)14 detected: Cytomegalovirus (CMV), HD, HSV type 1, HSV type 2 (HSV2), lymphogranuloma venereum, TP, and Varicella-Zoster Virus (VZV). The tests were performed following the manufacturer's instructions correctly, and amplification was performed in a CFX96 real-time thermocycler (Bio-Rad, Hercules, California, USA).

### Isolation of Neisseria gonorrhoeae

Urethral swabs collected for culture were placed in Amies transport medium with charcoal and transported to the reference laboratory within six hours of collection. Upon arrival, samples were inoculated onto selective Thayer-Martin agar and incubated at 35±1 °C in a humidified atmosphere enriched with 5 % CO_2_ or under anaerobic conditions. If no bacterial growth was observed after 24-hours, culture plates were reincubated for an additional 24-hours. Colonies presumptively identified as NG were subcultured onto chocolate agar for confirmation. Confirmed isolates were preserved in cryovials containing Casoy broth supplemented with 20 % glycerol and stored at −80 °C or in liquid nitrogen until further processing. Isolates were then transported to the Laboratory of Molecular Biology, Microbiology, and Serology at the Federal University of Santa Catarina (LBMMS/UFSC) in accordance with international regulations for transporting biological materials (UN3373, Category B).

### Antimicrobial susceptibility testing for Neisseria gonorrhoeae isolates

Minimum Inhibitory Concentrations (MICs) were determined using the agar dilution method in two consecutive rounds. In the first round, MICs for ceftriaxone, cefixime, azithromycin, ciprofloxacin, penicillin, and tetracycline were determined according to the Clinical and Laboratory Standards Institute (CLSI) guidelines. Testing occurred on GC agar base (Difco®, Becton, Dickinson and Company, Sparks, MD, USA) supplemented with 1 % Vitox® (Oxoid Ltd., Basingstoke, United Kingdom). Elevated MICs for ceftriaxone, cefixime, and azithromycin were validated using gradient diffusion strips (Etest®, bioMérieux), according to the manufacturer’s instructions. In the second round, two additional antimicrobials ‒ spectinomycin and gentamicin ‒ were incorporated, based on World Health Organization recommendations outlined in the Gonococcal Antimicrobial Surveillance Programme manual.^4^ MIC results were interpreted according to the criteria established by the Brazilian Committee on Antimicrobial Susceptibility Testing (BrCAST), which follows the European Committee on Antimicrobial Susceptibility Testing (EUCAST) breakpoints, ensuring international comparability of antimicrobial resistance data^5^ and classified as: (i) Susceptible, standard dosing (high chance of treatment success with standard regimens); (ii) Susceptible, increased exposure (high chance of success with higher drug exposure through dose adjustment or optimized concentration at the infection site); or (iii) Resistant (high chance of therapeutic failure even with increased exposure).

### Quality control and external validation

Quality control procedures were conducted using the NG reference strain ATCC 49,226 and at least three World Health Organization reference strains collected in 2008 (WHO F, G, K, L, M, N, O, and P) in each MIC determination. Each testing cycle was validated using four control strains, and acceptable MIC variation was defined as ±1 log₂ dilution unit relative to reference values. Beta-lactamase production was evaluated using nitrocefin discs (Becton, Dickinson and Company, Le Pont-de-Claix, France). To ensure external quality assurance, a representative subset of *N. gonorrhoeae* isolates was retested at the World Health Organization Reference Laboratory for Gonorrhea and Other Sexually Transmitted Infections (Örebro University, Sweden). The agreement between original and repeat MIC determinations (±1 log₂ dilution) was 99.05 %, confirming the reliability of antimicrobial susceptibility testing performed in Brazil.

### Statistical analysis

Descriptive analyses were performed using absolute and relative frequencies. Categorical variables were summarized as counts and percentages. Statistical analyses were conducted with SPSS version 16.0®, and analyses of antimicrobial resistance were carried out using R version 4.0.4. Associations between antimicrobial resistance profiles and the tested antimicrobials were evaluated using Fisher’s exact test, with a significance level of 5 % (α = 0.05).

### Ethical aspects

The study protocol was approved by the Institutional Ethics Committee (CAAE: 36844820.2.0000.5440) and authorized by the Municipal Health Secretariat of Ribeirão Preto. Written informed consent was obtained from all participants before enrollment. All patients received standard clinical care in accordance with the current Brazilian Clinical Protocol and Therapeutic Guidelines for Comprehensive Care for Sexually Transmitted Infections, regardless of study participation.^6^

## Results

A total of 144 samples were collected from 134 men aged 18- to 69-years (average age approximately 29-years), including 10 recurrent cases. Of the samples collected, 126 (87.5 %) were obtained from urethral discharge, and 18 (12.5 %) from genital ulcers. Molecular analysis of urethral discharge samples identified NG in 108 cases (85.7 %) and CT in 45 cases (35.7 %). Multiple cases of coinfection were observed, with simultaneous infection by CT and NG identified in 35 samples (28 % of the analyzed specimens). Two urethral discharge specimens contained four concurrent pathogens, one of them with NG, MH, MG, CT (2.1 %) and another with NG, MH, UP, CT (2.1 %), as illustrated in Table 1.

**Table 1:** Coinfections of urethritis and genital ulcers, Ribeirão Preto, SP, 2024.

Coinfection	Total (*n* = 144)	Urethral discharge (*n* = 126)	Genital ulcer (*n* = 18)
No	98 (68.06 %)	81 (64.29 %)	17 (94.44 %)
Yes	46 (31.94 %)	45 (35.71 %)	1 (5.56 %)
NG, CT	27 (58.7 %)	27 (60 %)	0 (0 %)
UU, NG, CT	5 (10.87 %)	5 (11.11 %)	0 (0 %)
NG, MG	3 (6.52 %)	3 (6.67 %)	0 (0 %)
UU, NG, MH	2 (4.35 %)	2 (4.44 %)	0 (0 %)
HSV-2, TP	1 (2.17 %)	0 (0 %)	1 (100 %)
MH, UP	1 (2.17 %)	1 (2.22 %)	0 (0 %)
NG, CT, TV	1 (2.17 %)	1 (2.22 %)	0 (0 %)
NG, MH, MG, CT	1 (2.17 %)	1 (2.22 %)	0 (0 %)
NG, MH, UP	1 (2.17 %)	1 (2.22 %)	0 (0 %)
NG, MH, UP, CT	1 (2.17 %)	1 (2.22 %)	0 (0 %)
UU, CT	1 (2.17 %)	1 (2.22 %)	0 (0 %)
UU, NG	1 (2.17 %)	1 (2.22 %)	0 (0 %)
UU, NG, UP	1 (2.17 %)	1 (2.22 %)	0 (0 %)

NG, Neisseria Gonorrhoeae; CT, Chlamydia Trachomatis; UU, *U. urealyticum*; MH, Mycoplasma Hominis; HSV-2, Herpes Simplex Virus type 2; TP, *Treponema Pallidum*; UP, *Ureaplasma Pauvum*; TV, Trichomonas Vaginalis.

Source – author, 2024.

**Fig. 1:**
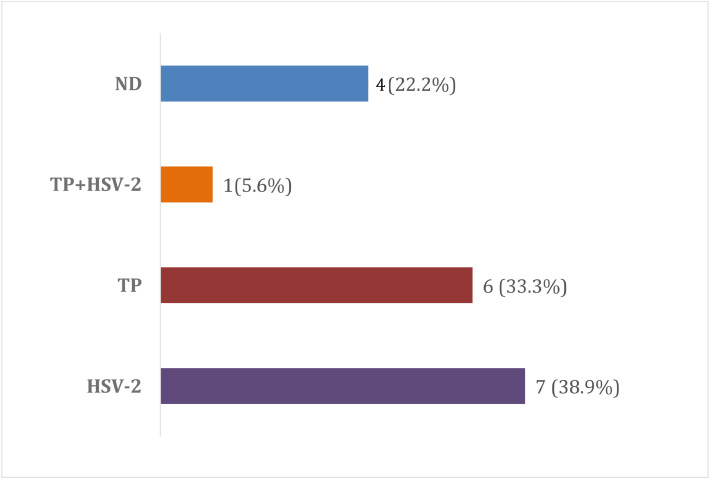
Etiological distribution of genital ulcer disease. ND, Not Determined (no pathogen identified using the diagnostic panel); HSV-2, Herpes Simplex Virus type 2; TP, *Treponema Pallidum*. Percentages represent the proportion of analyzed genital ulcer samples (*n* = 18).

The distribution of etiological agents identified in urethral discharge samples is summarized in Table 2. Of the 18 genital ulcer samples, no etiological agent was identified in 4 cases (22.2 %), this suggests that the cause may not be infectious, or the agent may not be among those identifiable by the molecular test used. In the remaining samples, HSV-2 was detected in 7 cases (38.9 %), TP in 6 cases (33.3 %), and one case showed coinfection (5.6 %). The etiological distribution of genital ulcers is shown in Fig. 1.

**Table 2:** Etiological profile of male urethritis identified by PCR (*n* = 126).

Identified pathogen	n (%)	95 % CI
*Neisseria gonorrhoeae*	108 (85.7)	(79.6‒91.8)
*Chlamydia trachomatis*	45 (35.7)	(27.4‒44.1)
*U. urealyticum*	11 (8.7)	(3.8‒13.7)
*Mycoplasma hominis*	6 (4.8)	(1.0‒8.5)
*Mycoplasma genitalium*	5 (4.0)	(0.6‒7.4)
*Ureaplasma parvum*	4 (3.2)	(0.1‒6.2)
*Trichomonas vaginalis*	1 (0.8)	(0.0‒2.3)

All urethral samples (*n* = 126) were tested for all pathogens included in the panel. Percentages represent the proportion of samples positive for each pathogen; multiple pathogens could be detected in the same sample.

Of the 108 urethral samples positive for NG, 98 isolates were available for antimicrobial susceptibility testing. Three isolates were excluded due to technical limitations associated with sample loss during processing, yielding a final dataset of 95 isolates with valid results. Susceptibility testing was performed for penicillin, ceftriaxone, ciprofloxacin, azithromycin, cefixime, spectinomycin, gentamicin, and tetracycline. All isolates were susceptible to ceftriaxone, cefixime, spectinomycin and azithromycin showed high susceptibility. In contrast, ciprofloxacin showed a high proportion of resistant isolates 67 (70.5 %), while penicillin 70 (73.7 %) and tetracycline 43 (45.3 %) showed substantial proportions of susceptible isolates with increased exposure, as well as resistant isolates. Detailed antimicrobial susceptibility results are presented in Table 3.

**Table 3:** Antimicrobial susceptibility profile of *Neisseria gonorrhoeae* isolates.

Antimicrobial agent	Number of isolates tested	Susceptible, n (%)	Susceptible, increased exposure, n (%)	Resistant, n (%)
**Penicillin G**	95	1 (1.0)	70 (73.7)	24 (25.3)
S (CIM ≤ 0.06 mg/L)				
SIE (CIM 0.125–1 mg/L)				
R (CIM 16 mg/L)				
**Tetracycline**	95	11 (11.6)	43 (45.3)	41 (43.1)
S (CIM ≤ 0.5 mg/L)				
SIE (CIM 1 mg/L)				
R (CIM > 1 mg/L)				
**Ciprofloxacin**	95	24 (25.3)	4 (4.2)	67 (70.5)
S (CIM ≤ 0.03 mg/L)				
SIE (CIM 0.06 mg/L)				
R (CIM < 0.06 mg/L)				
**Ceftriaxone**	95	95 (100.0)	–	–
S (CIM ≤ 0.125 mg/L)				
R (CIM > 0.125 mg/L)				
**Azithromycin**	95	84 (88.4)	–	11 (11.6)
S (CIM ≤ 1 mg/L)				
R (CIM > 1 mg/L)				
**Cefixime**	95	95 (100.0)	–	–
S (CIM ≤ 0.125 mg/L)				
R (CIM > 0.125 mg/L)				
**Spectinomycin**	95	95 (100.0)	–	–
S (CIM ≤ 64 mg/L)				
R (CIM > 64 mg/L)				
**Gentamicin[7]**	95	77 (81.0)	18 (19.0)	–
S (CIM ≤ 4 mg/L)				
SIE (CIM 4‒8 mg/L)				
R (CIM > 16 mg/L)				

Interpretation followed the Brazilian Committee on Antimicrobial Susceptibility Testing (BRCAST) criteria.

The number of isolates tested varied according to antimicrobial availability and testing period.

S, Susceptible; SIE, Susceptible, increased exposure; R, Resistant.

## Discussion

This study provides a local profile of the etiological causes of male urethritis and genital ulcer disease in southeastern Brazil. A substantial proportion of genital ulcer samples (24 %) lacked an identifiable etiological agent despite molecular diagnostic testing, consistent with findings from a nationwide molecular surveillance study in Brazil, which reported that approximately 28 % of genital ulcer samples lacked an identified agent.^3^ Similar proportions have been described in different geographic settings, where 27 % to 49.5 % of genital ulcer specimens remain etiologically undefined even when sensitive molecular techniques, such as multiplex PCR assays, are employed.^8^, ^9^, ^10^, ^11^ Etiologically negative genital ulcers are more common among sexually active men, especially men who have sex with men and those living with HIV, where HIV seroprevalence among cases of genital ulcer disease often exceeds 40 %–60 %.^9^^,^^10^^,^^12^^,^^13^ This scenario highlights the limitations of syndromic management in clinical practice and underscores the need to integrate laboratory-based diagnostics, HIV testing, and structured follow-up into routine STI care.

Among cases with a confirmed pathogen, the etiological profile of genital ulcer disease closely reflected the national pattern. HSV-2 remained the most frequently detected agent (38.9 %), similar to the 40.8 % observed in the nationwide study, reinforcing its primary role in the cause of genital ulcers in Brazil. TP was identified in 33.3 % of cases, slightly above the national estimate of 24.8 %, possibly due to local epidemiological differences, variations in healthcare-seeking behavior, or increased detection of early syphilis in this population. Coinfection with HSV-2 and TP was observed in one case (5.6 %), which is slightly higher than the national coinfection rate 4.4 %.^3^

Regarding urethral discharge samples, NG was the most frequently detected pathogen by PCR, followed by CT. Coinfections were common, particularly those involving *Chlamydia trachomatis* and *Neisseria gonorrhoeae*. These results align with global epidemiological data, as NG and CT remain the two most common bacterial sexually transmitted infections worldwide, responsible for approximately 82 million and 128 million new cases annually, respectively.^14^

Since the current study involved only men with symptomatic urethral discharge, NG was detected more frequently than CT. This agrees with epidemiological data indicating that NG is more often associated with symptomatic urethritis, while CT infections are usually asymptomatic and frequently go undiagnosed without active screening.^15^^,^^16^

We identified CT/NG coinfection in 28 % of the analyzed urethral specimens, a proportion substantially higher than the 14.3 % reported in the Brazilian nationwide etiological study.^3^ In an international context, our findings are consistent with data from public STI clinics in the United States, where CT/NG coinfection among patients treated for gonorrhea has been reported in approximately 20 % of men and up to 40 % of women.^17^ These data reinforce the importance of etiological testing and the need for combined antimicrobial coverage in the management of gonococcal urethritis.

Although the Brazilian national guideline recommends empiric treatment of urethritis with ceftriaxone plus a single dose of azithromycin as the first option,^6^ accumulating evidence shows lower microbiological cure rates of azithromycin for *Chlamydia trachomatis* compared with doxycycline.^18^, ^19^, ^20^

In this study, *Ureaplasma urealyticum* (UU), *Mycoplasma Hominis* (MH), and *Mycoplasma Genitalium* (MG) were found in 17.5 % of urethral specimens, with UU being the most common at 8.7 %, followed by MH at 4.8 %, and MG at 4.0 %. These findings indicate a modest yet clinically relevant contribution of atypical pathogens to the etiology of urethritis. Although infections caused by UU and MH are generally expected to respond to macrolide-containing regimens, increasing reports of resistance to this class of antimicrobials have been described.^21^^,^^22^ Of greater concern are MG infections, which are associated with rising antimicrobial resistance and treatment failure, particularly when treated with macrolides.^23^, ^24^, ^25^

The antimicrobial susceptibility profile observed in this study is consistent with global surveillance data, showing high resistance of NG to tetracyclines and fluoroquinolones, and preserved susceptibility to extended-spectrum cephalosporins.^26^ The absence of resistance to ceftriaxone and cefixime supports ceftriaxone as the preferred empiric therapy for gonococcal urethritis and underscores the need for continued local resistance surveillance. Although no resistance to spectinomycin or gentamicin was found in our isolates, the clinical use of aminoglycosides remains limited. This is due to historical treatment failures with spectinomycin as a monotherapy, its limited availability, and the absence of standardized susceptibility breakpoints and a reliable MIC-outcome correlation for gentamicin.^7^^,^^27^ Overall, our local susceptibility data support the use of cephalosporins as the primary treatment for gonococcal urethritis, with aminoglycosides reserved for special or rescue situations.

Among the study limitations, behavioral data should be noted. The medical records analyzed contained a substantial amount of missing information due to the lack of standardized documentation. Therefore, the study chose not to include variables related to sexual behavior, number of partners, condom use, and HIV status in the analysis. Another of the study’s limitations was the relatively small number of samples collected from genital ulcers compared to urethritis cases, owing to the use of convenience sampling based on the real-world clinical setting of the participating healthcare units. Additionally, no etiological agent was identified in some genital ulcer samples, reflecting the inherent limitations of available diagnostic methods.

## Conclusion

This study confirms NG and CT as the primary causes of male urethritis, while HSV-2 and TP predominate among cases of genital ulcer disease in this population. There were no available data regarding the prevalence and etiology of urethritis and genital ulcers in the male population in Riberião Preto. The high prevalence of coinfections underscores the importance of etiological diagnosis using molecular methods to guide specific treatment. From an antimicrobial resistance perspective, the absence of ceftriaxone resistance among NG isolates supports its continued use as first-line treatment, as per current national guidelines. Conversely, the continued presence of ciprofloxacin resistance underscores the need for ongoing local surveillance. Overall, these findings provide valuable epidemiological data and emphasize the importance of integrating laboratory-based diagnosis with antimicrobial resistance monitoring into STI control strategies.

## Authors' contributions

Felipe Barufaldi: Conceptualization; methodology; validation; formal analysis; data curation; investigation; writing-original draft; writing-review & editing. Maria Luiza Bazo: Conceptualization; methodology; validation; investigation; writing-review & editing. Jessica Motta Martins: Conceptualization; methodology; validation; investigation; writing-review & editing. Lis Aparecida de Souza Neves: Methodology; investigation; validation; writing-review & editing. Henrique Ciabotti Elias: Investigation; validation; data curation; writing-review & editing. Renata Karina Reis: Investigation; validation; data curation; writing-review & editing. Marcela Antonini: Investigation; validation; data curation; writing-review & editing. Rodrigo de Carvalho Santana: Conceptualization; methodology; validation; formal analysis; data curation; investigation; writing-original draft; writing-review & editing.

## Funding

This study was financed in part by the Coordenação de Aperfeiçoamento de Pessoal de Nível Superior (CAPES) – Brasil (Finance Code 001).

## Data availability

The data that support the findings of this study are available from the corresponding author upon reasonable request.

## Conflicts of interest

The authors declare no conflicts of interest.
